# Supplementary material for: Understanding degraded speech leads to perceptual gating of a brainstem reflex in human listeners
Source: PLoS Biol. 2021 Oct 20;19(10):e3001439. doi: 10.1371/journal.pbio.3001439 (PMC8559948; doi:10.1371/journal.pbio.3001439)
Supplement: Attachment — Submitted filename: response-to-reviewers.docx [file pbio.3001439.s011.docx]

Editorial Points

1) we think reviewer 1 makes a good point about your title. You should be more specific unless you can make a clear demonstration otherwise.
2) You will also note that reviewer 3 suggests you could split this submission into several manuscripts, to decompress the message. We think you should keep all the information within this paper, as its impact relies on the comprehensive collection of data and analyses.

We appreciate the editorial comments and we have edited the manuscript accordingly.

**Reviewer #1:** This study investigates cochlear, brainstem and cortical responses during passive and active listening monaurally. The active listening task consisted of detecting non-words in a single stream of words and non-words, and the passive task involved presenting similar speech streams while participants were watching and attending to a stop-motion movie. Cochlear and brainstem responses during the two tasks were monitored by recording otoacoustic emissions (OAEs) and auditory brainstem responses (ABRs) to clicks presented to the ear contralateral to speech ear. Cortical responses were obtained by analyzing the P1, N1, N2, N400 and the late positivity component (LPC) after each word. The speech was degraded by noise-vocoding and by adding babble (BN) and speech-shaped noise (SSN).

A main finding is that cochlear responses are inhibited (OAEs suppressed) during active but not passive listening to vocoded speech, while they are inhibited during passive but not active listening to masked speech. This finding is interpreted as indicating that medial olivo-cochlear (MOC) efferents are gated selectively when their activation helps to perform the task, i.e., they are activated when actively listening to vocoded speech because their activation enhances the amplitude fluctuations in vocoded speech, which facilitates understanding vocoded speech; conversely, they are activated in passive listening (during the active visual task) because MOC efferent activation suppresses the speech+noise stimulus that is irrelevant to perform the active visual task. The authors reason that MOC efferents are not active during active listening to masked speech because their activation would not improve the representation of speech in simultaneous noise. To support this interpretation, the authors use a computational model of the auditory brainstem with MOC efferent control. The model predicts better speech-envelope coding with than without MOC efferent activation for vocoded speech but not for masked speech.

Another main finding is that brainstem (wave V) responses are stronger in active than in passive listening to masked speech, while they are not different in the active and passive listening tasks when listening to vocoded speech. Furthermore, in active listening, cortical responses (P1, P2 and LPC) are stronger when listening to masked speech than when listening to vocoded speech. These findings are interpreted as reflecting an engagement of the central auditory system in de-noising masked speech but not when listening to vocoded speech in quiet.

Combined, the findings are interpreted as reflecting that central/cortical auditory areas are involved in de-noising when listening to speech in noise, while MOC efferents are gated during tasks where participants benefit from the effects of MOC efferent activation.

The data are interesting and the interpretations thought-provoking. There are several issues, however, that prevent me from recommending this study for publication in its present form. I would be happy to reconsider my recommendation once the authors ponder (and hopefully address) the following concerns and comments.

Major issues

**Reviewer 1-major issue 1**.- The authors should be more careful in using the term MOC reflex. The term reflex is conventionally used to refer to an activation of MOC efferents by sound. Therefore, it is confusing to see the same term used to refer to an activation of MOC efferents by active listening (i.e., by attention). Noting the difference in terminology might help reconciling present and past findings. It is conceivable, for example, that MOC-efferent activation by sound (i.e., the MOC reflex) facilitates the neural representation of speech in noise, but attention-gated activation of MOC-efferents facilitates task performance. Therefore, I suggest carefully revising the terminology throughout the manuscript for accuracy and consistency with previous studies.

*Response to Reviewer 1-major issue 1:* We appreciate this comment and have made a distinction between the MOC reflex, its constituent MOC fibers and their reflexive activation vs. top-down gating in the introduction (Lines 47-50 and 53-56). Nevertheless, our non-invasive measure of inner ear activity (the suppression of click-evoked otoacoustic emissions) is an indirect metric of overall MOC reflex activity. This includes both its reflexive sound-evoked activation and top-gating by higher auditory centres (via possible direct excitation/inhibition and/or modulation of reflexive activity). We have therefore used “MOC reflex” in the remainder of the manuscript for two reasons: 1) We have conducted our experiments in awake and behaving humans, where speech was both the contralateral activator of the MOC reflex and the basis for a lexical decision task, therefore, we can only infer from our experimental design whether MOC reflex activity is being perceptually gated on or off and 2) We consider using a simple, consistent terminology throughout the manuscript (i.e., referring to the MOC reflex and its reflexive activation vs. top-down gating) as important to avoid confusion for a general audience.

**Reviewer 1-major issue 2**.- The manuscript title does not accurately reflect the content and scope of the study and is misleading. As it stands, it implies that the MOC reflex facilitates the understanding of any speech (in noise), when in fact the data do not support this idea. The data support that activation of MOC efferents facilitate understanding noise-vocoded speech in quiet, but not necessarily speech in general, and certainly not speech embedded in noise. I advise revising the title for accuracy.

*Response to Reviewer 1-major issue 2:* We appreciate this comment and in consequence we have edited the title: “Understanding degraded speech leads to perceptual gating of a brainstem reflex in human listeners”.

**Reviewer 1-major issue 3**.- The description of the stimuli is not clear. It is unclear if words were presented with or without silent gaps between them. Also unclear is whether the BN and SSN were continuous during the 12-minute experimental blocks, including any silent gaps between words, or if they were gated with each word (as implied in L643). Activation of the MOC efferents (and their effects on CEOAEs) would have been very different for continuous and gated noises. Because of the relatively slow time-course of MOC efferent activation (~280-300 ms), if noises were gated with words, and there were silent gaps between words, then MOC efferents would have been active only during the last portion of each word. If my understanding is correct, this was the certainly case for noise-vocoded speech but it is unclear if it was also the case for noisy speech. The temporal course of the various stimuli should be explained more clearly for this reviewer to be able to interpret the results. I suggest including a supplementary figure illustrating more clearly the time course of words, BN or SSN, clicks, and recording windows of OAEs, ABRs, and cortical responses.

*Response to Reviewer 1-major issue 3:* We appreciate this observation. As the reviewer noticed noises and words were gated on/off together, therefore both the speech and the noise had the same duration with a 3 secs inter-stimulus interval (button press for the active listening condition (lexical decision task) or no button press just silent during passive listening task). Therefore, noise vocoded and masked speech had the same duration, hence comparable MOC reflex contributions across different speech conditions. We have edited Figure 1, so it includes the time-course of all stimuli and physiological measurements (revised Figure 1 A-C).

This issue is also of concern to the model simulations as we presented the same speech tokens individually as inputs to our auditory periphery model. This is one of several reasons that, in our new (revised) simulations, we have applied a fixed attenuation of cochlear gain in conditions were efferent feedback was included (see Results section “**Simulated MOC reflex improves the neural representation of vocoded speech, but not speech-in-noise**”(Lines 260-261) and Methods section “**Implementation of efferent feedback in simulations**” (Lines 918-931). As a result, the MOC reflex’s activation time course/magnitude did not vary between speech tokens and their degradation. We acknowledge the possibility that sluggish activation of the MOC reflex (as modelled in our previous simulations) may influence the size of any effect of the MOC reflex on envelope encoding. However, ensuring a constant MOC attenuation across all speech tokens (both natural and degraded) provides a more controlled investigation of the direction of this effect. We discuss the MOC reflex’s time course in the section titled, “**Considerations when modelling the MOC reflex”** (Lines 665-686).

**Reviewer 1-major issue** **4**.- The writing needs to be improved. I appreciate the direct and concise writing style, but the manuscript contains a lot of information, some of which is hard to follow without further details. Specific instances where the text can be improved are given below.

*Response to Reviewer 1-major issue 4:* We appreciate these observations we have edited the manuscript following the Minor/specific comments below.

**Reviewer 1-major issue** **5**.- The rationale for using the various stimulus configurations is not explained. For example, what was the purpose of (or even better, the hypothesis for) using vocoded speech? Without further details, it seems as though they are building a story backwards from the findings. Please explain upfront the rationale for your stimuli, in particular, the purpose/hypothesis of using vocoded speech in quiet.

*Response to Reviewer 1-comment 5:* We appreciate this observation. We have now clarified the rationale for using our stimulus configurations in the Introduction. “Unlike speech-in-noise, noise-vocoded speech allows for manipulation of intelligibility without the addition of spectrally broadband acoustic energy that intrinsically activates the MOC reflex. Noise-vocoded speech should therefore enable better detection of any modulation of the MOC reflex by perceptual gating as task difficulty increases” (Lines 81-84).

**Reviewer 1-major issue** **6**.- The interpretation of the findings is not rounded and not 100% warranted by the data. Why would MOC efferent activation not occur during passive listening to vocoded speech? In this condition, the task was to visually attend to the silent movie and the vocoded speech was irrelevant. Therefore, participants would have also benefited from the MOC system inhibiting the irrelevant auditory stimulus as they benefited (according to your interpretation) from the MOC inhibiting the masked speech in the passive auditory task.

*Response to Reviewer 1-comment 6:* We appreciate this comment and we have included a rationale for the absence of CEOAEs suppression during the passive listening of noise vocoded speech in the Discussion section. “… auditory stimuli may capture attention in a different manner depending on how easy they are to detect: i.e., saliency-based (bottom-up processes). For example, the pitch conveyed in the fundamental frequency, F0, here in terms of the voice pitch, is a highly salient cue that plays an important role in the perceptual segregation of speech sources. When speech is noise-vocoded, saliency carried by the envelope periodicity of the speech is diminished. In the context of our passive conditions, then, it is possible that noise-vocoded speech was not salient (or distracting) enough to elicit a reduction in cochlear gain sufficient to suppress irrelevant auditory information when attention was presumably focused elsewhere (i.e., on watching a silent film).” (Lines 503-511),

**Reviewer 1-major issue** **7**.- The model support to your findings is not 100% convincing for three reasons. First, it is based on the assumption that the speech understanding is based solely on the neural representation of the speech envelope. This assumption may or may not be true, but people can understand speech reasonably well with only fine structure (Lorenzi et al., 2006, PNAS). Second, if I understood correctly, the correlation between neural and stimulus envelope representations is based on neural responses at one CF only (?), which is unlikely the case. Third, when the same model (or a version of it) has been used in combination with an automatic speech recognizer, the model predicts MOC efferent activation to improve the recognition of speech in noise (Brown et al., 2010, JASA; Yasin et al., 2018, JASA; Yasin et al., 2020, IEEE Access). This latter aspect is discussed in the manuscript but is dismissed by the authors. Altogether, I find that the model and the chosen metric used to predict speech recognition (Delta_rho_ENV) are used in a limited (and somewhat biased) way.

*Response to Reviewer 1-comment 7*.1 The purpose of our model simulations was to seek a potential explanation for the stimulus-dependence of CEOAE suppression we observed experimentally. By focusing on stimulus envelope cue, we do not claim to be able to predict overall speech understanding or even match current/previous behavioural data quantitively. We acknowledge temporal fine structure as an important cue for speech understanding in the Results section and include supporting information showing that adding efferent feedback improves the encoding of fine structure information for the masked speech conditions (S6 Figure and Lines 364-371). This result is important as it suggests that our CEOAE data for BN5 and SSN3 cannot be explained by considering phasic information in low frequencies alone. For noise-vocoded stimuli, we did not measure TFS encoding given that scrambled TFS cues due to noise-vocoding would ensure values of ρTFS that are 0 or near zero (Heinz and Swaminathan, 2009). However, Moon et al. (2014) showed that emphasis on the ρENV best predicted human behaviour for noise-vocoded stimuli such as used here.

*Response to Reviewer 1-comment 7*.2 As regards the frequencies channels tested, we have expanded the range (from 7 frequencies between 1 and 2kHz) to 30 frequencies between 0.1 and 4.5kHz in our revised simulations (as in Brown et al., 2010). Our results demonstrate consistent, stimulus-dependent effects on envelope encoding in both high (> 1.5kHz) and low (< 1.5kHz) frequency bands. Given our focus on comparing neural envelopes of degraded speech with their natural counterparts in the auditory nerve, across-channel comparisons of neural envelopes for the three manipulations, i.e., calculating ρENV between frequency channels for each speech manipulation (Swaminathan and Heinz, 2011), were not the immediate focus of our simulations and would be more relevant to describing envelope processing in higher auditory centres.

*Response to Reviewer 1-comment 7*.3 As regards our analysis, *sumcors* allowed us to identify and isolate the neural response components associated with the stimulus envelope so we could test our hypothesis that envelope encoding of noise-vocoded and masked stimuli was differentially affected by including efferent feedback. In contrast, an automatic speech recognizer (ASR) would not have allowed us to test this hypothesis, as it is difficult to identify how the ASR weights stimulus cues when classifying speech (Brown et al., 2010, JASA). Furthermore, the studies mentioned by the reviewer using the MAP models alongside an automatic speech recognizer have only quantified improvements to speech-in-noise recognition with efferent feedback and therefore cannot explain our experimental observations that the MOC reflex was gated off when subjects actively performed the lexical decision task for speech-in-noise (BN5 and SSN3, Figure 2F). We extensively discussed this as well as difference between the instantiations of the MAP and MAP_BS models in a new section titled, **“Reconciling stimulus-specific effects of the MOC reflex with previous studies using MAP and efferent feedback”** (Lines 628-663).

Minor/specific comments
**Reviewer 1-minor/specific comment 1:** L18. The reflex does not really extract anything, though it might help extracting the salient features in question. Please rephrase for accuracy.

*Response to Reviewer 1-minor/specific comment 1:* We have edited the phrase accordingly (Line 18)

**Reviewer 1-minor/specific comment 2:** L23. The author(s) of the model reported exactly the opposite, i.e., they reported that MOC efferent activation improved the recognition of speech in noise by an automatic speech recognizer.

*Response to Reviewer 1-minor/specific comment 2* This phrase refers to our own results. We do discuss however the differences between our models and those that have preceded in the Discussion section, **“Reconciling stimulus-specific effects of the MOC reflex with previous studies using MAP and efferent feedback”** (Lines 628-663).

**Reviewer 1-minor/specific comment 3:** L50-51. The end of this sentence appears to be incomplete.

*Response to Reviewer 1-minor/specific comment 3:* Thank you for noticing, we have completed the sentence (Lines 52-53).

**Reviewer 1-minor/specific comment 4:** L55. Here and elsewhere, it would be appropriate to cite the review of Lopez-Poveda (2018) Front Neurosci.

*Response to Reviewer 1-minor/specific comment 4*: We have cited Lopez-Poveda (2018) accordingly (Line 58 and 565).

**Reviewer 1-minor/specific comment 5:** L62. Replace the semicolon with a comma.

*Response to Reviewer 1-minor/specific comment 5:* Replaced (Line 65)

**Reviewer 1-minor/specific comment 6:** L63. Remove the space in "OAE s".

*Response to Reviewer 1-minor/specific comment 6:* Removed as part of rewriting.

**Reviewer 1-minor/specific comment 7:** L79. I am not sure what mean by "homologous visual and auditory scenes". In what sense were they homologous?

*Response to Reviewer 1-minor/specific comment 7:* We have replaced “homologous” with “identical” and edited the sentence for clarity. (Line 90)

**Reviewer 1-minor/specific comment 8:** L82. For clarity, I suggest starting this paragraph with "We found that…".

*Response to Reviewer 1-minor/specific comment 8:* Edited accordingly (Line 92).

**Reviewer 1-minor/specific comment 9:** L85. It would be clearer to say "assessed in terms of the suppression of CEOAEs"?

*Response to Reviewer 1-minor/specific comment 9:* Edited accordingly (Lines 95-96).

**Reviewer 1-minor/specific comment 10:** L94. "levels of listening effort". Are you saying (in this paragraph) that listening to vocoded speech involves spending different levels of effort than listening to speech in noise? I would say that it is not only "different levels of" but also "different kinds of" effort?

*Response to Reviewer 1-minor/specific comment 10:* The statement has been rewritten to reflect the suggested edits (Lines 106-107).

**Reviewer 1-minor/specific comment 11:** L101. "to 'natural' speech (i.e., speech-in-quiet)". This is confusing. Consider rephrasing as "to 'natural' speech in quiet".

*Response to Reviewer 1-minor/specific comment 11*: Rephrased as requested (Lines 113-114).

**Reviewer 1-minor/specific comment 12:** L105. "Three levels of task difficulty". This description is confusing because, actually, I see more than three levels. Considering rephrasing for clarity/accuracy.

*Response to Reviewer 1-minor/specific comment 12:* Changed to: “Distinct levels of task difficulty” (Lines 117-118).

**Reviewer 1-minor/specific comment 13:** L108. "This was statistically confirmed". What was confirmed?

*Response to Reviewer 1-minor/specific comment 13*: It has been clarified that the levels of task difficulty were statistically confirmed (Lines 120-121).

**Reviewer 1-minor/specific comment 14:** L113-115. The last statement appears to be incomplete. Please revise.

*Response to Reviewer 1-minor/specific comment 14*: Statistics have been reordered to make more sense (Lines 124-127).

**Reviewer 1-minor/specific comment 15:** L120. What test was applied?

*Response to Reviewer 1-minor/specific comment 15*: we have specified now that a t-test was applied (Lines 132)

**Reviewer 1-minor/specific comment 16:** L122-123. I am not sure what the statistical test indicated at the end of the sentence refers to.

*Response to Reviewer 1-minor/specific comment 16*: We now specify that a One-way ANOVA was used for this analysis (Lines 135).

**Reviewer 1-minor/specific comment 17:** L132. It would be useful to describe what d' refers to, so that the reader can understand the legend and figure without having to read the Methods. Also, the "white circles" referred to are not visible (or present?) in the figure. Perhaps, this is because of the poor quality (low resolution) of the figures provided to the reviewers.

*Response to Reviewer 1-minor/specific comment 17*: It has been specified in Figure 1 legend that: d’ is a measure of accuracy calculated as: Z (correct responses) – Z (false alarm) (i.e., Z (correct responses) = NORMSINV (correct responses)) (Lines 149-150). The visibility of the white circles noting the mean d’, has been corrected.

**Reviewer 1-minor/specific comment 18:** L160. "auditory efferent activity". Do you mean efferent to the cochlea or more generally?

*Response to Reviewer 1-minor/specific comment 18*: We have specified that we are referring to attentional gating of the MOC reflex (Lines 182-183).

**Reviewer 1-minor/specific comment 19:** L162. I am not sure (at this point in the text) how iso-performance was achieved. Please explain.

*Response to Reviewer 1-minor/specific comment 19*: We have specified that iso-performance here referred to comparable levels of lexical discrimination achieved across speech manipulations (Lines 184-185).

**Reviewer 1-minor/specific comment 20:** L168. "(Figure 2A)" I think you probably mean "(Figure 1C)". Please double check and correct it if appropriate.

*Response to Reviewer 1-minor/specific comment 20*: Corrected (Line 191).

**Reviewer 1-minor/specific comment 21:** L193. Delete "and".

*Response to Reviewer 1-minor/specific comment 21*: Deleted

**Reviewer 1-minor/specific comment 22:** L194. "(Figure 1C)" I think you mean "(Figure 1D)". Please double check and correct it if appropriate.

*Response to Reviewer 1-minor/specific comment 22*: Corrected (Line 222)

**Reviewer 1-minor/specific comment 23:** L198. "differing results" to what results are your referring to?

*Response to Reviewer 1-minor/specific comment 23*: We have clarified that we were referring to the stimulus-dependence of inner ear and brainstem responses (Lines 225-226).

**Reviewer 1-minor/specific comment 24:** L208. "gain changes are sufficient"; sufficient for what? Please explain.

*Response to Reviewer 1-minor/specific comment 24:* We have edited this statement. “While this is evident for both listening conditions in masked speech, the similarity of ABR magnitudes in the midbrain for active and passive listening of noise-vocoded stimuli is indicative of feed-forward amplification that compensates for reduced cochlear gain during active listening.” (Lines 236-239)

**Reviewer 1-minor/specific comment 25:** L215-217. Alright, but other authors have shown this not to be only effect of MOC efferent activation. See my major comments. Why not use, for example, the model in combination with a speech recognizer?

*Response to Reviewer 1-minor/specific comment 25:* We chose to compare stimulus envelope encoding between natural and degraded stimuli as it is the cue most clearly shared between all three degraded stimuli and their natural counterparts (particularly relevant for noise-vocoded stimuli in quiet, Moon et al., 2014). We have however included evidence that fine structure encoding in low frequency channels (< 1.5kHz) is actually improved with efferent feedback for speech-in-noise (S6 Figure) therefore suggesting it cannot explain our CEOAE data for BN5 and SSN3 stimuli in the active listening conditions (Figure 1E and 2F). As regards the automatic speech recognizer, it would not have allowed us to disentangle the stimulus cues being weighted during classification. Furthermore, there is no evidence to suggest that the automatic speech recognizer performs identification using the same cue weighting as humans. As mentioned above, the results of previous studies suggest that the automatic speech recognizer would not be able to explain our CEOAE data for masked speech. Please see Discussion section, **“Reconciling stimulus-specific effects of the MOC reflex with previous studies using MAP and efferent feedback”** (Lines 628-663) for more details.

**Reviewer 1-minor/specific comment 26:** L220-222. Speech recognition is unlikely based on information carried by LSR units? Why not apply the same analyses to all fiber types?

*Response to Reviewer 1-minor/specific comment 26*: Simulations for HSR fibers were also performed at the same 75 dB intensity (S4 Figure) and their importance to envelope encoding at low and high intensities are alluded to in the Results (Lines 351-363).

**Reviewer 1-minor/specific comment 27:** L244. Do you really mean 400 fibers/1ms bin-width or 400 stimulus presentations per fiber? It is not the same thing.

*Response to Reviewer 1-minor/specific comment 27:* Thanks for noticing this, we have specified that we were referring to the responses of 400 fibers binned at 1ms (Line 291).

**Reviewer 1-minor/specific comment 28:** L279-281. How does this compare to the findings of past studies that used the same model? See my general comments.

*Response to Reviewer 1-minor/specific comment 28:* Of the numerous studies using the MAP model to test efferent feedback (Brown et al., 2010; Clark et al., 2012; Yasin et al., 2020), Clark et al (2012) have used the most similar stimulus degradation to the current study i.e., speech-in-babble noise. Including efferent feedback when presenting digits in babble noise at either +5 or + 10 SNR improved speech recognition by 10 to 60%. However, as the SNR was decreased to 0 or negative values then the improvement tended towards zero. When testing time constants of dynamic efferent feedback, Yasin et al. (2020) also found that improvements in speech recognition were SNR-dependent. They consistently found peak improvement was observed, independent of the MOC reflex’s time constant, for +10 SNR stimuli. This fits neatly with the results of our revised simulations as we observe a mean improvement in envelope encoding for speech-in-babble noise at + 10 SNR with the MOC reflex (Results, Lines 343-347, and Discussion, Lines 615-617).

**Reviewer 1-minor/specific comment 29:** L282-291 and more generally. Did the masking noise in the model and the experiments start sufficiently well before the words to guarantee full activation of MOC efferents at the time when words started and during the full duration of the words?

*Response to Reviewer 1-minor/specific comment 29:* We presented the same speech tokens used in our behavioural tests as inputs to our auditory periphery model. As a result, noise and signal were gated on and off simultaneously for the speech-in-noise conditions. In our revised simulations, we have applied a fixed attenuation of 15dB attenuation of cochlear gain, ensuring consistent efferent feedback (both activation time courses/magnitudes) across speech tokens and their different degradations [Methods section “**Implementation of efferent feedback in simulations**” (Lines 918-931)]

**Reviewer 1-minor/specific comment 30:** L310-316 and more generally. Were the BN and SSN such that the MOC *reflex* (i.e., an activation of MOC efferents by sound) be active during passive listening in noise (Fig. 1C)?

*Response to Reviewer 1-minor/specific comment 30*: Unfortunately we do not understand how the quoted lines refer to comment. However we have discussed activation of the MOC reflex by noise maskers in Lines 520-528, emphasising in the revisions that this activation is reflexive (Line 521).

**Reviewer 1-minor/specific comment 31:** L320-321. Why are so many electrodes needed to record the responses shown in Fig. 3? Why are responses shown only for the active listening task? What were responses like (i.e., did they show any significant features) in the passive auditory task (active visual task)?

*Response to Reviewer 1-minor/specific comment 31:* Event related activity from 13 electrodes were analysed (Figure 3 and S7 Figure): (FZ (1), F3(2), F4(3), CZ (4), C3(5), C4(6), TP7(7), TP8(8), T7(9), T8(10), PZ (11), P3(12), P4(13) due to their relevance in attentional and language brain networks: (Kutas and Federmeier, 2000, Kutas and Federmeier, 2011; Davenport and Coulson, 2013; Broadway et al., 2015, Trimmel et al., 2017). We have included the reasoning behind electrodes selection in Figure 3 and S7 Figure legends. Responses for the hardest/least intelligible conditions are shown in main results section (Figure 3), because we were testing the specific hypothesis of different cortical engagement across speech manipulations despite similar overall task performance. In S7 Figure, all grand-average ERPs for Active vs. Passive conditions are shown as well as briefly discussed in the Discussion (Lines 694-696).

**Reviewer 1-minor/specific comment 32:** L356. Delete "2016)".

*Response to Reviewer 1-minor/specific comment 32*: Deleted

**Reviewer 1-minor/specific comment 33:** L373. "preserves cochlear gain to prevent deterioration of envelope encoding". Why would activation of MOC efferents deteriorate envelope encoding in quiet or in noise?

*Response to Reviewer 1-minor/specific comment 33:* We appreciate this observation. Our CEOAE and modelling data suggests that envelope encoding of speech in quiet is improved when the MOC reflex is activated. However, when referring to the absence of MOC reflex activation during active listening to speech-in-noise we have rephrased our statement, so instead of “deteriorate” we suggest that the activation of MOC reflex does not contribute to envelope encoding (Lines 461-464).

**Reviewer 1-minor/specific comment 34:** L381. Close the brackets after "as speech".

*Response to Reviewer 1-minor/specific comment 34*: Edited (Line 472)

**Reviewer 1-minor/specific comment 35:** L391. "to maintain iso-performance" ◊ "possibly to maintain iso-performance".

*Response to Reviewer 1-minor/specific comment 35*: Edited (Line 482)

**Reviewer 1-minor/specific comment 36:** L407. "where participants" ◊ "when participants". (?)

*Response to Reviewer 1-minor/specific comment 36*: Edited (Lines 499-500)

**Reviewer 1-minor/specific comment 37:** L412-413. "required to attention". Please revise this text.

*Response to Reviewer 1-minor/specific comment 37*: Edited: “required to attend” (Line 514).

**Reviewer 1-minor/specific comment 38:** L417-428. This whole paragraph is hard to follow. Ideas are thrown without indicating which aspects of the data support the ideas in question.

*Response to Reviewer 1-minor/specific comment 38:* We have rewritten this section with clear reference to the relevant data (Lines 518-534). In addition, we clarified what top-down Vs bottom-up mechanisms are which is also pertinent to the reviewer’s first major comment regarding use of the term, the MOC reflex.

**Reviewer 1-minor/specific comment 39:** L435. The cited study [62] not only suggests that MOC reflex expands interaural cues associated with sound localization but also; it suggests that the MOC enhances amplitude modulations and suppresses the noise (and thus enhances the SNR) in the ear with the better acoustic SNR (see for example, Fig. 2 in Lopez-Poveda et al., 2020, Ear Hear).

*Response to Reviewer 1-minor/specific comment 39*: Thank you for drawing our attention to this. We have included this here (Lines 540-541) and also added it to the list of references supporting the MOC reflex’s role in the “unmasking” of signal in noise (Lines 563).

**Reviewer 1-minor/specific comment 40:** L469. It would be appropriate to also cite Marrufo-Pérez et al. (2021). Front Neurosci.

*Response to Reviewer 1-minor/specific comment 40*: Reference added (Line 572)

**Reviewer 1-minor/specific comment 41:** L532, "that this analysis" what analysis exactly?

*Response to Reviewer 1-minor/specific comment 41:* We have rewritten this section and clarified predicted effects of testing noise-vocoded speech in the ASR in Discussion section, **“Reconciling stimulus-specific effects of the MOC reflex with previous studies using MAP and efferent feedback”** (Lines 651-664)

**Reviewer 1-minor/specific comment 42:** L614. Please define Z.

*Response to Reviewer 1-minor/specific comment 42*: Z has been defined in Figure X legend (Z (correct responses) = NORMSINV (correct responses)) as well as in the Methods section (Lines 776-777).

**Reviewer 1-minor/specific comment 43:** L643. Was the BN (and SSN) gated with each word? If so, this may have been insufficient for a full activation of the MOC reflex by the noise. Or were the noises continuous? See my general comments.

Response to Reviewer 1-minor/specific comment 43: Both noises (BN and SSN) where gated on and off with each word so both the noise and speech had the same duration. We have clarified this in the Methods (BN: Lines 811-813 and SSN: 817-819) and Figure 1.

**Reviewer 1-minor/specific comment 44:** L682. In what units was CEOAE magnitude expressed? In dB SPL or in Pascal?

*Response to Reviewer 1-minor/specific comment 44*: CEOAE magnitudes were expressed in dB SPL, this has been clarified in Figure 1 legend (Line 161) and Methods section (Lines 861-862)

**Reviewer 1-minor/specific comment 45:** L682. CEOAE_baseline (first 60 sec). I am confused. You just said that baseline CEAOE magnitude was calculated over the first and last 60 sec.

*Response to Reviewer 1-minor/specific comment 45:* Thanks for noticing this. Two baseline periods were initially recorded, but due to artifacts (participant’s movements in the 2^nd^ baseline recording) only the first minute had the required quality for analysis (SNR ≥ 6 dB and with > 80% of epochs retained). This has been clarified in the Figure 1 legend (Lines140 -141) and Methods section: “**CEOAEs acquisition and analysis**” (Lines 853-856).

**Reviewer 1-minor/specific comment 46:** L689-694. This (long) sentence appears to be incomplete (missing a verb?). The MEMR could have been activated by the speech for a significant proportion of participants (see Feeney et al., 2017, Ear Hear).

*Response to Reviewer 1-minor/specific comment 46:* Thanks for noticing that the MEMR activation in relation to experimental stimuli levels needed further clarification. We have included a new section: “**Middle ear muscle reflex assessment and stimuli calibration**” in the Methods (Lines 747-764) where we address in detail how MEMR was assessed and how all experimental stimuli levels were kept below participant’s MEMR threshold activation.

**Reviewer 1-minor/specific comment 47:** L696. I am not sure what the 10-20 system is. Please explain.

*Response to Reviewer 1-minor/specific comment 47*: It has been clarified that: “EEG data were acquired according to the 10–20 system (internationally standardized scalp electrodes placement (Homan 1998)”, in the methods section (Line 868).

**Reviewer 1-minor/specific comment 48:** L698. I am not sure what LSB in nV/LSB refers to. Please define LSB.

*Response to Reviewer 1-minor/specific comment 48*: It has been clarified that LSB in nV/LSB refers to the Least Significant Bit (Line 870).

***References for reviewer 1 comments***

Heinz MG, Swaminathan J. Quantifying envelope and fine-structure coding in auditory nerve responses to chimaeric speech. J Assoc Res Otolaryngol. 2009;10: 407–423.

Moon IJ, Won JH, Park M-H, Ives DT, Nie K, Heinz MG, et al. Optimal combination of neural temporal envelope and fine structure cues to explain speech identification in background noise. J Neurosci. 2014;34: 12145–12154.

Swaminathan, J., & Heinz, M. G. (2011). Predicted effects of sensorineural hearing loss on across-fiber envelope coding in the auditory nerve. The Journal of the Acoustical Society of America, 129(6), 4001-4013.

Brown GJ, Ferry RT, Meddis R. A computer model of auditory efferent suppression: implications for the recognition of speech in noise. J Acoust Soc Am. 2010;127: 943–954.

Clark NR, Brown GJ, Jürgens T, Meddis R. A frequency-selective feedback model of auditory efferent suppression and its implications for the recognition of speech in noise. J Acoust Soc Am. 2012;132: 1535–1541.

Yasin I, Drga V, Liu F, Demosthenous A, Meddis R. Optimizing Speech Recognition Using a Computational Model of Human Hearing: Effect of Noise Type and Efferent Time Constants. IEEE Access. 2020;8: 56711–56719.

Kutas M, Federmeier KD. Thirty years and counting: finding meaning in the N400 component of the event-related brain potential (ERP). Annu Rev Psychol. 2011;62: 621–647.

Trimmel K, Sachsenweger J, Lindinger G, Auff E, Zimprich F, Pataraia E. Lateralization of language function in epilepsy patients: A high-density scalp-derived event-related potentials (ERP) study. Clin Neurophysiol. 2017;128: 472–479.

Kutas M, Federmeier KD. Electrophysiology reveals semantic memory use in language comprehension. Trends Cogn Sci. 2000;4: 463–470.

Davenport T, Coulson S. Hemispheric asymmetry in interpreting novel literal language: an event-related potential study. Neuropsychologia. 2013;51: 907–921.

Broadway JM, Franklin MS, Schooler JW. Early event-related brain potentials and hemispheric asymmetries reveal mind-wandering while reading and predict comprehension. Biol Psychol. 2015;107: 31–43.

Homan RW, Herman J, Purdy P. Cerebral location of international 10-20 system electrode placement. Electroencephalogr Clin Neurophysiol. 1987;66: 376–382.

**Reviewer #2:** The present manuscript presents a large data set to suggest that top-down modulation of the cochlea efferent system depends on the stimulus material, and in particular on how the envelope of speech stimuli is modulated by cochlear gain. This is interesting, as a number of previous studies have suggested top-down control of the efferent system, but the direction of the modulation has been variable, such that the role of this mechanism is not yet well understood. The present manuscript might therefore help to resolve some of the variance between previous studies. The experiments are overall well done and the manuscript is well written. I have only a few questions, which, however, I think are important to interpret the data. As I could not find a certain answer to these in the manuscript, I will discuss them below:

**Reviewer 2-comment (1)** Based on Figure suppl. 1B, there is a 3-s interstimulus interval (ISI) between speech tokens (elsewhere I read that the speech tokens were concatenated?).

In the natural speech and NV conditions, I assume the ISI is silent. However, how is the temporal shape of the masker in the other conditions? In the methods section on page 26, it is mentioned that maskers were 60-s long (l. 642), but then also that the speech token and masker segments were matched in duration (l. 643), but no details about onset/offset ramps are provided. Thus, I wasn't sure if the duration of the masker is only of the duration of each speech token, or if it continuous through a 3-sec ISI. In the latter case, this would have important influence on the CEOAE, because the ongoing masker would lead to stronger suppression than the silence in the other condition, which would in fact match the data. First, the stimulus setup needs to be explained in more detail (methods refer to Fig Supp 6, but this only includes the spectra). If there are really ISIs that are silent versus filled with a masker, this would need to be considered in the explanation of the data and in the model calculations.

*Response to Reviewer 2-comment 1*: We appreciate this observation. Noises and words were gated on/off together, therefore, both noise vocoded and masked speech manipulations incorporate 3-sec inter-stimulus intervals which are silent. Therefore MOC-efferent contributions are comparable across different speech degradations. We have edited Figure 1, so it includes time-course details of all stimuli and physiological measurements (revised Figure 1 A-C). In addition, we have revised our simulations, using a fixed attenuation of 15dB, independent of word duration or spectro-temporal content, to represent an active MOC reflex. This ensures a constant MOC attenuation across all natural and degraded speech tokens, providing a more controlled comparison of neural envelopes with and without MOC reflex [see Methods section “**Implementation of efferent feedback in simulations**” (Lines 918-931)]. We now discuss the importance of the MOC reflex’s time course in the section “**Considerations when modelling the MOC reflex.”** (Lines 665-686)

**Reviewer 2-comment (2)** The model suggests that the presence of the MOC reflex enhances envelope coding measured by pENV in the range of 3.5%. How can this effect be compared with CEOAE suppression and modulation of wave V? On first glance the effect size appears relatively small. Could the authors provide some arguments how the effect size could be compared between these measures?

*Response to Reviewer 2-comment 2:* We appreciate this comment and in consequence we have now reported the effect size for all our measurements (behavioural, physiological and modelling). Although as the reviewer suggested, the effect sizes might look small, all calculations of standardized effects sizes for significant effects ranged from medium to large. Therefore, we are confident that the effects observed in our data are not only reliable but also biologically meaningful. Comparing standardized effect sizes of variables measured in different scales (pENV, CEOAEs, ABRs) has been described as problematic because the standardized effect size only represents the intrinsic variability withing a given measurement (Lakens, 2013). It will need to be transformed from standardized effect sizes to a common effect size index (using meta-analysis approach) which goes beyond the scope of our study and its goals.

**Reviewer 2-comment (3)** For the early part of the cortical response, the waveform appears to start right at 0 ms latency, in particular for the conditions where a masker was used. Again, it would be helpful to know more details about the timing of the masker to interpret these waveforms. Is there a common onset of masker and speech token, or is the masker ongoing? (The authors write that "the speech and noise tokens overlap", but this would be the case both with continuous as well as gated maskers). In the case of gated maskers, the onset would represent a mixture of masker and speech token.

*Response to Reviewer 2-comment 3:* We appreciate that the relationship between the masker and speech is confusing in the manuscript. We have therefore modified Figure 1 (A-C) to show more clearly the time course of the masked speech relative to the cortical responses. As suggested by the reviewer, there was indeed a common onset of maskers and speech tokens (see Methods, BN: Lines 811-813 and SSN: 817-819).

***Reference for reviewer 2 comments***

Lakens D. Calculating and reporting effect sizes to facilitate cumulative science: a practical primer for t-tests and ANOVAs. Front Psychol. 2013;4: 863.

Reviewer #3: Review of "Perceptual gating of a brainstem reflex facilitates speech understanding in human listeners" by Hernandez-Perez et al.

This manuscript describes a study aimed at determining the extent to which Medial Olivocochlear (MOC) reflex activity depends on active/passive listening during the presentation of speech that was intrinsically degraded (i.e., vocoded speech) or degraded by the addition of background noise. The effect of presenting vocoded or noise-degraded speech on the amplitudes of click-evoked otoacoustic emissions (OAEs) and the auditory brainstem response (ABR) was measured by presenting a continuous click train to the non-test ear. Late-latency evoked potentials were also measured in response to the vocoded or noise-degraded speech presented in the test ear. All measurements (OAEs, ABR, late-latency potentials) were obtained simultaneously while the subject actively detected the presence of non-sense words or passively watched a stop-motion movie. The primary findings are 1) suppression of OAEs in response to vocoded speech was significantly larger during active versus passive listening, 2) suppression of OAEs in response to speech degraded by steady-state noise was significantly larger during passive versus active listening, 3) ABR-wave V amplitudes obtained during the presentation of speech-shaped noise were significantly larger during active versus passive listening, and 4) cortical potential amplitudes measured during active listening were larger for noise-degraded speech compared to vocoded speech. The authors interpret a subset of these findings in the context of a computational auditory model where emphasis was placed on analyzing changes in envelope coding for simulations that did versus did not simulate the MOC reflex.

This study is well designed and appears to produce results that are consistent and are interpretable in the context of previous literature. I appreciate the approach to include simultaneous measurement of perception, OAEs, and event-related potentials and I feel this approach is a great strength to the design of the study. Although I am unable to identify any fatal flaws with the study, I have some general comments that I wish to express to the authors - some of which I give as a matter of opinion and not as changes I require before recommending the manuscript for publication.

GENERAL COMMENTS
**Reviewer 3-comment 1**: The rationale for the methods surrounding the model simulations is underdeveloped. The manuscript refers to the "Matlab Auditory Periphery and Brainstem" (MAP-BS) computational model; however, a citation is not provided for this model. I was unable to determine 1) whether the authors were proposing a new (unpublished) model, 2) why currently published computational models with efferent feedback were not considered (e.g., Messing et al., 2009; Brown, Ferry, and Meddis, 2010; Smalt et al. 2014), 3) why the MAP-BS model is an improvement over previous models, 4) how the predictions from the MAP-BS model may be similar to or different from previously published models.

*Response to Reviewer 3-general comment 1*: Thank you for this comment, it has led to several changes to our modelling: 1. we have implemented the MAP-BS model with a fixed attenuation of cochlear gain in the active MOC reflex conditions, as in Brown et al. (2010); 2. A new reference is included citing a study that also used the MAP-BS model (Moezzi et al., 2014); 3. we have described more clearly how the model works in the methods/results (Figure 2A and Lines 902-931). We have discussed the similarities and differences between MAP and MAP_BS models as well as between *sumcor* and automatic speech recognition analyses in a section titled, **“Reconciling stimulus-specific effects of the MOC reflex with previous studies using MAP and efferent feedback”** (Lines 628-664).

In short, the MAP_BS model shares much code with MAP models used by Brown et al., 2010 and later Clarke et al., 2012 and Yasin et al., 2020 . However where the MAP-BS model mainly differs from its predecessor is that it can only generate stochastic spike trains as output (Sumner et al ., 2002) (as opposed to the MAP model which is typically used to generate spiking probability across all fibers simulated [Meddis, 1986]). It is these spikes trains that allows us to extract the neural envelope by comparing AN responses to normal and polarity-inverted stimuli in short (50 µs) time windows (see sumcor analysis in the methods). In the revised simulations, we use a fixed 15dB attenuation of cochlear gain in the active MOC reflex condition (observed to be near optimal for stimuli of similar intensity by Brown et al., 2010). This aligns our model further with the MAP model used by Brown et al. whilst also applying the most up-to-date biophysical parameters in the MAP model family for spike train generation (Moezzi et al., 2014). Using a fixed attenuation of cochlear suppression offers a more controlled comparison of neural envelopes with and without efferent feedback [see Methods section “**Implementation of efferent feedback in simulations**” (Lines 918-931)]. As a result, we can make simpler (and more general) conclusions about what effect an active MOC reflex has on envelope encoding.

As regards the automatic speech recognizer (ASR), we have included a section titled, **“Reconciling stimulus-specific effects of the MOC reflex with previous studies using MAP and efferent feedback”** (Lines 628-664) to discuss the differences between *sumcor* and ASR analyses and why we used the former in this study. In short, we were specifically interested in qualifying whether efferent feedback offered a benefit or not to envelope encoding for different word degradations (rather than maximizing/quantifying improvements to speech recognition as in previous studies [Brown et al., 2010; Clark et al., 2012; Yasin et al., 2020]). Using an ASR would not have allowed us to disentangle the stimulus cues being weighted during classification as it exploits any available cue to identify the digit sequence correctly. Furthermore, given the previous results for speech-in-noise [as well as the decreasing digit recognition for clean, “natural’ speech in quiet with the MOC reflex (Brown *et al*., 2010)] (Brown *et al.*, 2010; Clark *et al*., 2012; Yasin *et al.*, 2020), an ASR would most likely not have explained our CEOAE data for active listening to any of our speech manipulations.

**Reviewer 3- general comment 2**: The level of the vocoded and noise-degraded speech was likely intense enough to elicit the middle ear muscle (MEM) reflex. Although the authors measured clinical thresholds for the MEM reflex, there is evidence that clinical-based thresholds are, on average, 18.5 dB higher than more sensitive measures of the MEM reflex based on power reflectance (Feeney and Keefe, 2001). The discussion does not address the likelihood that the stimuli used in the experiment stimulated the MEM reflex; nor does the discussion address how this stimulation modifies the interpretation of the results.

*Response to Reviewer 3- general comment 2:* Thanks for noticing that the MEMR activation in relation to experimental stimuli levels needed further clarification. We have included a new section: “**Middle ear muscle reflex assessment and stimuli calibration**” in the Methods (Lines 747-764) where we address in detail how MEMR was assessed and how all experimental stimuli levels were kept below participant’s MEMR threshold activation. In short, we implemented modified clinical protocols (wide-band measures of reflectance), as described by Feeney and Keefe (2001), to accurately measure individual MEMR thresholds. As all participants’ thresholds were above 75db HL, we conclude that there is very little likelihood that we have stimulated the MEMR during stimulus presentation (at 75dBA for speech or 75dB p-p for clicks).

**Reviewer 3- general comment 3**: The manuscript introduces an appreciable amount of new information and findings in a relatively restricted length. I feel this may be overwhelming for the reader. I consider myself an expert in psychophysics, auditory modelling, and MOC efferents, and despite this expertise I found the manuscript overwhelming to read and digest. I feel there are two and maybe three papers stuffed into this one manuscript. For example, if the model simulations are the result of a new (unpublished) model, I think it would be appropriate to dedicate a separate paper to more fully describing the model and the interpretation of the results in the context of the model. Similarly, I am unaware of previous studies that evaluated contralateral suppression of OAEs using vocoded stimuli in passive versus active listening tasks. The discussion of such findings in the context of temporal envelope coding is very dense and may be more digestible if the experimental results were presented in a separate manuscript. The discussion of such a manuscript could more carefully relate these results to suppression of OAEs measured with other stimuli with modulated envelopes and in tasks with/without active listening. I give this final comment as an option for the authors to consider rather than a requirement before I recommend the manuscript for publication.

Messing, D. P., Delhorne, L., Bruckert, E., Braida, L. D., & Ghitza, O. (2009). A non-linear efferent-inspired model of the auditory system; matching human confusions in stationary noise. Speech communication, 51(8), 668-683.
Brown, G. J., Ferry, R. T., & Meddis, R. (2010). A computer model of auditory efferent suppression: implications for the recognition of speech in noise. The Journal of the Acoustical Society of America, 127(2), 943-954.
Smalt, C. J., Heinz, M. G., & Strickland, E. A. (2014). Modeling the time-varying and level-dependent effects of the medial olivocochlear reflex in auditory nerve responses. Journal of the Association for Research in Otolaryngology, 15(2), 159-173.
Feeney, M. P., & Keefe, D. H. (2001). Estimating the acoustic reflex threshold from wideband measures of reflectance, admittance, and power. Ear and hearing, 22(4), 316-332.

*Response to Reviewer 3- general comment 3*: We appreciate these comments and suggestions. However, we have been advised by the editorial board not to separate the manuscript as the impact of our work comes from the number of paradigms and analysis employed. Hopefully after all these constructive revisions, the manuscript will be more accessible.

OTHER COMMENTS
**Reviewer 3-other comments 1:** Line 28 (abstract): it would help to quickly state/summarize these strategies.

*Response to Reviewer 3-other comments 1*: Line 28 in the abstract has been rewritten to state the strategies (Lines 29-31).

**Reviewer 3-other comments 2:** Line 31: I think the goal is not "cocktail-party" listening per se, but effective/robust "cocktail-party" listening.

*Response to Reviewer 3-other comments 2:* L31 has been rewritten as requested (Line 33)

**Reviewer 3-other comments 3**: Lines 50-51: something is wrong with this sentence

*Response to Reviewer 3-other comments 3:* We appreciate this comment. It appears that our reference manager botched some of the references. We have therefore edited this sentence (Line 53).

**Reviewer 3-other comments 4:** Line 57: consider softening the statement by using "unclear" instead of "controversial"

*Response to Reviewer 3-other comments 4*: Edited as recommended (Line 59).

**Reviewer 3-other comments 5:** Line 62: Be more explicit. OAE amplitudes are not always reduced by contralateral acoustic stimulation. In some cases, as with DPOAEs, the amplitude may increase.

*Response to Reviewer 3-other comments 5:* Thanks for this comment. This section has been rewritten to indicate that OAE magnitudes are typically reduced when activating the MOCR (Line 65).

**Reviewer 3-other comments 6:** Line 63: There is an extra space between the "E" and "s" of OAEs.

*Response to Reviewer 3-other comments 6*: Edited as part of rewriting (Lines 66-68)

**Reviewer 3-other comments 7:** Line 63-64: This sentence should state that the magnitude of OAE suppression may relate to speech perception performance. (not just OAE magnitude).

*Response to Reviewer 3-other comments 7:* Sentence has been rewritten to reflect this (Lines 66-68).

**Reviewer 3-other comments 8:** Line 64-65: Efferent modulation of cochlear gain could depend…? (I'm not sure what is meant by "confounding effects.")

*Response to Reviewer 3-other comments 8:* Edited as suggested (Line 68-70).

**Reviewer 3-other comments 9:** Line 108: State what "this" is for clarity. (e.g., This "modulation of task difficulty" was statistically…)

*Response to Reviewer 3-other comments 9:* Edited as suggested (Line 120)

**Reviewer 3-other comments 10:** Lines 155-156: Why did the direction of the t-statistic change? Shouldn't all t-statistics be in the same direction if CEOAE suppression is present?

*Response to Reviewer 3-other comments 10:* Thanks for noticing this, there is indeed an incorrect (-) sign in the t-test reported for the natural condition, the correct t-test now shows: natural: [t (24) = 2.33, p = 0.03, d=0.50] (Lines 176-177).

**Reviewer 3-other comments 11:** Lines 179-180: "Effects" is vague. Do you mean intermediate magnitude of suppression of OAEs?

*Response to Reviewer 3-other comments 11:* This statement has been edited to refer to a “mild suppression of CEOAE for speech masked by babble noise” (Lines 203-204).

**Reviewer 3-other comments 12:** Lines 207-210: Or alternatively, the changes in ABR are inherited from the cochlear reduction in gain in the noise-degraded speech conditions (brainstem passively transmits signal from lower auditory centers), and for vocoded stimuli there is a compensatory central amplification to offset the reduction in cochlear gain.

*Response to Reviewer 3-other comments 12:* We thank you for the suggestion and we have adjusted these lines (Lines 235-241) to reflect the reviewer’s interpretations.

**Reviewer 3-other comments 13:** Lines 485-488: This conclusion may depend on SNR. For favourable SNRs the expansion would enhance the effective speech envelope while releasing AN fibers from adaptation/saturation produced by the noise.

*Response to Reviewer 3-other comments 13:* We also consider this an important point and have presented two conditions per stimulus type: Voc8 and Voc16 for noise-vocoded; BN5 and BN10 for babble noise and SSN3 and SSN8 for speech-shaped noise (Figure 2E and S4 Figure B). The reversal of the MOC reflex’s effect for BN5 to BN10 (from impairment to improvement of envelope encoding on average) highlights this point (Lines 343-349) and we discuss this point further in a new section, **“Dependence of MOC reflex on signal-to-noise ratio”** (Lines 597-626).

**Reviewer 3-other comments 14**: Lines 496-501: This sentence is long and hard to read. Consider breaking it apart.

*Response to Reviewer 3-other comments 14*: This long sentence has been divided into two as suggested (Lines 606-611).

**Reviewer 3-other comments 15:** Line 597: State that all subjects did not complete all experimental measurements.

*Response to Reviewer 3-other comments 15*: Edited as suggested (Lines 743-744).

**Reviewer 3-other comments 16:** Line 599: Close parentheses after "tympanometry."

*Response to Reviewer 3-other comments 16*: Parentheses closed (Line 745)

**Reviewer 3-other comments 17:** Line 600: Close parentheses that started before "assessed" on line 599.

*Response to Reviewer 3-other comments 17:* Parentheses closed (Line 746)

**Reviewer 3-other comments 18:** Line 600-601: Something is wrong with this sentence.

*Response to Reviewer 3-other comments 18:* Thanks for noticing this. This sentence refers to the middle ear muscle assessment. Since this assessment has required clarification from most of the reviewers, we decided to add a section in the methods were all the details about the MEMR assessment and stimuli levels have been addressed: “**Middle ear muscle reflex assessment and stimuli calibration**” (Lines 747-764). Therefore, the referred sentence has been edited and integrated into this section.

**Reviewer 3-other comments19:** Line 620: Why was the speech material only from one talker? Please address whether the results of the experiment would be different if a different talker were used. Can we be confident that the results would be similar with a different talker?

*Response to Reviewer 3-other comments 19:* Thanks for pointing out this detail about our paradigm. Our speech comprehension task was a lexical decision paradigm that is known to be a task that is easy to administer and relatively quiet for EEG and OAE recording purposes (participants only had to press a button when detecting a non-word rather than having to repeat words or sentences). To perform this task here in Australia, we needed a corpus of Australian-English spoken words and non-words. The most up-to-date version of this speech material (Australian version of the CNC corpus and its non-words counterparts (used in clinical audiology)) is only available with one Australian female speaker.

In terms of how performance might change were a mixture of different talkers used, including males, it has been reported that normal hearing individuals achieve similar levels of speech understanding for either female or male speakers, with a slight advantage towards the male speaker (0.7 dB SNR Larsby et al., 2015). In consequence, the use of a female talker might have slightly increased the overall task difficulty. However, this may have been compensated by the familiarity of the single speaker’s voice that has been shown to increase speech intelligibility in noise (Holmes et al., 2018; Holmes et al., 2021). Rather than being a caveat, we would argue that using a single speaker simulates a more realistic environment as it more closely represents a one-to-one conversation scenario.

Although it has been shown that physiological measurements can be used to differentiate female and male voices by most likely picking up differences in pitch cues among speakers (Latinus & Taylor 2011; Ding & Simon 2013; Saiz-Alia et al., 2021), the effects of attending or not to a talker has not been reported as differing depending on the talker’s gender (Teder et al., 1993; Coch et al., 2005; Etard et al., 2019). Moreover, reports of convergence in the long-term average speech spectrum (LTASS) across different languages and speakers (10 females and 10 males) (Byrne et al., 1994) suggests that when a sufficient variability of talkers is sampled, differences between female and male speakers are abolished. Therefore, we do not foresee any significant impact of using male talkers or a mixture of different speakers when evoking attention-driven modulation of the MOC reflex.

**Reviewer 3-other comments 20:** Line 729: Why were the model simulations limited to these frequencies? I expected the simulations would include a broad range of CFs given that the stimulus of interest (speech) is broadband.

*Response to Reviewer 3-other comments 20:* Thank you for the suggestion. In our new (revised) simulations, we have now expanded the frequency range of our AN fiber from 0.1 to 4.5 kHz (30 logarithmically-spaced channels) as in Brown et al. (2010). Although we separate our results into two frequency bands (above and below 1.5 kHz) (see Figure 2E) to highlight the effects of the MOC reflex on envelope encoding at higher frequencies, we observe the same stimulus-specific effects of the MOC reflex on envelope encoding in both bands.

**Reviewer 3-other comments 21:** Line 750: The model is not fully described in the text. For example, does the model simulate basilar membrane nonlinearity (i.e., suppression, compression, level-dependent tuning)? I assume it does, but I could not find the description in the text.

*Response to Reviewer 3-other comments 21:* Thank you for the comment, the model used in our study directly descends from Meddis’ MAP models and therefore includes a dual resonance nonlinear filterbank to simulate outer/middle ear stages. We describe this more extensively in the methods sections **“Auditory Nerve Simulations”** (Lines 902-917) and “**Implementation of efferent feedback in simulations**” (Lines 918-931) as well as providing a schematic for the model in Figure 2A.

***References for reviewer 3 comments***

Clark NR, Brown GJ, Jürgens T, Meddis R. A frequency-selective feedback model of auditory efferent suppression and its implications for the recognition of speech in noise. J Acoust Soc Am. 2012;132: 1535–1541.

Yasin I, Drga V, Liu F, Demosthenous A, Meddis R. Optimizing Speech Recognition Using a Computational Model of Human Hearing: Effect of Noise Type and Efferent Time Constants. IEEE Access. 2020;8: 56711–56719.

Moezzi B, Iannella N, McDonnell MD. Modeling the influence of short term depression in vesicle release and stochastic calcium channel gating on auditory nerve spontaneous firing statistics. Front Comput Neurosci. 2014;8. doi:10.3389/fncom.2014.00163.

Sumner CJ, Lopez-Poveda EA, O’Mard LP, Meddis R. A revised model of the inner-hair cell and auditory-nerve complex. J Acoust Soc Am. 2002;111: 2178–2188.

Meddis R. Simulation of mechanical to neural transduction in the auditory receptor. J Acoust Soc Am. 1986;79: 702–711.

Larsby, Birgitta, et al. "The influence of female versus male speakers' voice on speech recognition thresholds in noise: Effects of low-and high-frequency hearing impairment." Speech, Language and Hearing 18.2 (2015): 83-90.

Holmes, Emma, Grace To, and Ingrid S. Johnsrude. "How Long Does It Take for a Voice to Become Familiar? Speech Intelligibility and Voice Recognition Are Differentially Sensitive to Voice Training." Psychological Science (2021): 0956797621991137.

Holmes, E., Folkeard, P., Johnsrude, I. S., & Scollie, S. (2018). Semantic context improves speech intelligibility and reduces listening effort for listeners with hearing impairment. International Journal of Audiology, 57(7), 483-492.

Latinus, Marianne, and Margot J. Taylor. "Discriminating male and female voices: differentiating pitch and gender." Brain topography 25.2 (2012): 194-204.

Ding, Nai, and Jonathan Z. Simon. "Robust cortical encoding of slow temporal modulations of speech." Basic Aspects of Hearing. Springer, New York, NY, 2013. 373-381.

Saiz-Alía, Marina, Peter Miller, and Tobias Reichenbach. "Otoacoustic emissions evoked by the time-varying harmonic structure of speech." Eneuro 8.2 (2021).

Teder, Wolfgang, Teija Kujala, and Risto Näätänen. "Selection of speech messages in free-field listening." Neuroreport: An International Journal for the Rapid Communication of Research in Neuroscience (1993).

Coch, Donna, Lisa D. Sanders, and Helen J. Neville. "An event-related potential study of selective auditory attention in children and adults." Journal of cognitive neuroscience 17.4 (2005): 605-622.

Etard, Octave, et al. "Decoding of selective attention to continuous speech from the human auditory brainstem response." NeuroImage 200 (2019): 1-11.

Byrne, Denis, et al. "An international comparison of long‐term average speech spectra." The journal of the acoustical society of America 96.4 (1994): 2108-2120.

**Reviewer #4:** Hernandez-Perez et al review

This study uses a clever experimental design with simultaneous OAE, ABR and speech EEG recordings to investigate how cochlear gain changes when (modified) speech is presented in attended vs unattended listening. The topic is very relevant and timely, because little is known about how bottom-up and top-down mechanisms alter the MOC reflex when listening to (degraded) speech. This study is important in that it evaluates cochlear, brainstem and late potential changes simultaneously, which is a rare but necessary approach to understand the cascade of peripheral and neural adjustments the MOC reflex can inflict. In the study, CEOAE gain changes are interpreted as MOC reflex changes that appear to be modulated by the speech modifications and listening state. Model simulations with MOC reflexes on or off can capture the effect of how the stimulus modifications affect the envelope similarity between the natural-MOC condition. The paper ends with a theoretical interpretation of how the trends in the data can be interpreted in a model of (attention-driven) top-down and bottom-up MOC modifications that depend on the acoustics.

Major comments:
The analysis of the results is of very high quality. However, I think that the connection with the ABR results can be made stronger and that the model/interpretation proposed is not necessarily the "sole" or "conclusive" answer as to how these results could have arisen. At the same time, there is a methodological reference issue (baseline for CEOAE was different from that in modelling) that may have had an impact on the results interpretation. This would have to be sorted out before we can fully grasp the meaning of the results and evaluate whether the proposed models are appropriate. It is rather a collection of aspects that make me doubt whether the data observations warrant the final conclusions, but hopefully these can be addressed/mitigated/clarified in a revision.

The below comments are in random order of importance:

Minor spelling:
**Reviewer 4-minor spelling 1**: L33: its => their

*Response to Reviewer 4-minor spelling 1*: Corrected (Line 37).

**Reviewer 4-minor spelling 2**: L46: check em-dash and bracket positions

*Response to Reviewer 4-minor spelling 2:* Corrected (Lines 48-49)

**Reviewer 4-minor spelling 3**: L51: check bracket text and references

*Response to Reviewer 4-minor spelling 3:* Corrected (Line 53)

**Reviewer 4-minor spelling 4**: L58: "the/a" listening task

*Response to Reviewer 4-minor spelling 4*: Corrected (Line 61).

**Reviewer 4-minor spelling 5**: L63: OAEs

*Response to Reviewer 4-minor spelling 5*: Corrected in rewrite (Line 66)

**Reviewer 4-minor spelling 6**: L120: "three-way" ANOVA?

*Response to Reviewer 4-minor spelling 6*: The ANOVAs performed in this section were all one-way ANOVAs with one Factor (Stimuli-Type), this has been clarified. (Line 133).

**Reviewer 4-minor spelling 7**: L168: should be figure 1, no?

*Response to Reviewer 4-minor spelling 7*: Corrected (Line 191)

**Reviewer 4-minor spelling 8**: L180: CEOAEs

*Response to Reviewer 4-minor spelling 8*: Corrected (Line 204)

**Reviewer 4-minor spelling 9:** L193: "and was", change into "- was"

*Response to Reviewer 4-minor spelling 9*: Corrected (Line 221)

**Reviewer 4-minor spelling 10:** L194: Figure 1D?

*Response to Reviewer 4-minor spelling 10*: Corrected (Lines 121-122)

**Reviewer 4-minor spelling 11:** L199: Vs. => vs.

*Response to Reviewer 4-minor spelling 11:* Corrected here and elsewhere (line 226)

**Reviewer 4-major comment 1:** L202-205: Question related to testing intrinsic differences in the two populations, and inference of conclusion: "the differences observed in auditory brainstem/midbrain activity and cochlear gain can be attributed to the presented speech degradation when there is no relationship between CEOAE suppression and ABR amplitudes in the natural speech condition" L202-205 show results of a t-test where wave-III/V amplitudes [in microV] are compared to CEOAE suppression [in dB] to support this statement statistically. Does it make sense to compare dB values to microV amplitudes, they have different units and why not characterize ABR reduction rather than amplitude to have a meaningful comparison? At the moment, the 0-hypothesis of your t-test is: "there is no difference in the distributions: dB suppression vs ABR amplitude in microV". To me, such a comparison makes no sense at all, there might never be a theoretical relationship between these two units. Have I misunderstood something about the purpose of the test you conducted, or can you use the same units?

*Response to Reviewer 4-major comment 1:* Thanks for pointing out that this section needed clarification. This section aimed to test the null hypothesis that the two populations of normal hearing participants (Population 1-noise vocoded experiment and Population 2-masked conditions) were not intrinsically different. For this we compared common conditions obtained in both data sets Population 1-CEOAEs to natural speech vs. Population 2- CEOAEs to natural speech). This analysis was extended to the ABRs, therefore CEOAEs were not compared to ABRs. We have clarified this comparison in the manuscript (Lines 225-234).

**Reviewer 4-major comment 2:** Figure 1C-D, It is confusing to see CEOAE suppression in negative dBs, are these just small values or are you observing a "release from suppression", i.e. more gain in the system when more background noise is presented?

*Response to Reviewer 4-major comment 2:* Thanks for pointing out that the y axis in our Figure 1C is confusing. This is however a common way to label CEOAE suppression visually (Giraud et al., 1997; Mertes et al., 2019) however we have clarified in the figure legend that negative values describe increased CEOAE suppression relative to baseline (Lines 161-162).

**Reviewer 4-major comment 3.1:** L206-210 It is hard to follow your conclusion here, you would need to motivate this better. When I look at the passive conditions only for CEOAE suppression (Figure 1C), and compare the two natural conditions, there is quite some variability between the degree of CEOAE suppression, what is the reason for this, and is this meaningful?

*Response to Reviewer 4-major comment 3.1.* Thanks for pointing this out. We believe this is closely related to **Reviewer 4-major comment 1** where the section exploring intrinsic differences between populations was not stated clearly enough in the text. When comparing CEOAE suppression between populations (natural in the vocoded experiment vs. natural in the masked conditions), it did not reach statistical significance either for the passive or the active listening conditions. Therefore, we concluded that the patterns of CEOAE suppression observed in the different experiments are not due to differences between the populations tested but the actual stimuli being processed (Lines 225-234).

**Reviewer 4-major comment 3.2:** My worry is that the BN10/BN5 conditions show more suppression than the corresponding natural condition, but not to the natural condition that came with the vocoded manipulations. Hence it may be difficult to conclude the "reduction of the cochlear gain for masked stimuli" part of your sentence. You then go on to write that the "ABR magnitudes appear only inherited from [this cochlear gain reduction]". Again, only looking at the passive conditions (Figure 1D), I can see that wave-V is reduced for the BN & SSN compared to the vocoded (significance not sure) conditions, but you do not compare against the "Natural" condition. This collapsing in Figure 1D is confusing to me, at least you'd have to isolate the "Natural" conditions. L208-210 are even more difficult to follow, it is not clear how exactly the results lead you to conclude this.

Response to Reviewer 4-major comment 3.2. Regarding CEOAE suppression when speech in babble noise was presented, we only observed mild effects, with CEOAEs significantly smaller than their own baseline measures during passive listening (shown in the planned t-test, S1 Table 2). However, when performing an rANOVA for this experiment comparing active vs. passive listening conditions, no statistical differences were observed, therefore this prevented us to explore stimuli differences such as natural vs BN5 or BN10 as the reviewer is suggesting. That is to say that since no group effect was found, we did not perform any post-hoc tests. As a result, we have inferred that intermediate effects were observed for the babble noise experiment. This is clarified in Lines 179-185 and Lines 203-206.

**Reviewer 4-major comment 3.3**. You then go on to write that the "ABR magnitudes appear only inherited from [this cochlear gain reduction]". Again, only looking at the passive conditions (Figure 1D), I can see that wave-V is reduced for the BN & SSN compared to the vocoded (significance not sure) conditions, but you do not compare against the "Natural" condition. This collapsing in Figure 1D is confusing to me, at least you'd have to isolate the "Natural" conditions. L208-210 are even more difficult to follow, it is not clear how exactly the results lead you to conclude this. L208-210 are even more difficult to follow, it is not clear how exactly the results lead you to conclude this.

*Response to Reviewer 4-major comment 3.3.* When performing an rANOVA analysis for the ABRs, only main effect of conditions (active vs. passive) was statistically significant. Hence, the reason why only main effects are shown in Figure 1E and mentioned in Lines 219-223. The lack of significance for Stimuli main effect or a significant interaction Conditions x Stimuli, prevented us to explore any post-hoc differences between natural vs BN10 or BN5.

**Reviewer 4-major comment 4.** L228: Here, logically you use the "natural speech" as the template for comparison with the iso-response conditions, why not do the same for the CEOAE and ABR conditions? There you are referring to the control as the condition without speech. It would be more consistent to use the same reference throughout the paper. In consequence, CEOAE suppression might become positive in some cases, but it would make it easier to judge the effect of speech manipulations, attention and masking if you used the natural speech as reference in all cases.

*Response to Reviewer 4-major comment 4:* Our experimental paradigm was designed to maintain fixed levels of task difficulty. This allowed us to preserve comparable task relevance across the speech manipulations and therefore avoid confounding effects of task difficulty on top-down modulation of MOC reflex activation. Our statistical design (rANOVA) tested the specific hypothesis of a task difficulty effect (interaction between Conditions X Stimulus-type) in our data. This design, therefore, includes natural speech as the easiest speech intelligibility condition. This is the reason why natural speech cannot be assumed to be a baseline, specifically considering that not even ceiling effects are observed in our behavioural data, see Figure 1D.

In line L228 (of the original Manuscript), we are not testing a task difficulty hypothesis in the model but instead testing the hypothesis that suppression of cochlear gain can differentially affect neural encoding of the stimulus envelope for noise-vocoded and masked speech (Lines 257-258). To achieve this, we needed target neural envelopes (responses to clean/Natural speech) against which to compare the neural envelope for the speech manipulations. This analysis of the model therefore provides a potential rationale (based on encoding of the stimulus envelope cue) for the pattern of our CEOAE results.

**Reviewer 4-major comment 5.** L236: It is unclear to me what you mean by "to their natural speech controls" in this section, because earlier on you write "Natural speech control simulations were always performed with the MOC reflex". From a visual inspection of Fig.2A, where this distinction does not matter, VOC8 resembles well the corresponding Nat (compare MOC with MOC and AN only with AN only). The BN/SNN matches less well the corresponding natural conditions (compare MOC with MOC, and AN with AN). The MOC on or off does not change much to these trends as long as you use the corresponding Nat condition. So yes, VOC shows increased similarity to Nat than BM/SNN, but this appears MOC independent. Adding MOC does have an effect on all conditions, but from a visual inspection, but the trends are similarity in comparison to the Nat condition appear the same. Perhaps you did refer to corresponding controls for these comparisons, but it is not clear to me from the description, and the used reference may have a big influence on the results interpretations.

*Response to Reviewer 4-major comment 5:* As suggested, we have also calculated ρENV_AN_ using the ‘corresponding’ Nat condition (i.e. Nat_ANonly,_) natural speech without the MOC reflex (S3 Figure and Lines 328-335). Despite the visual similarity between the PSTHs of normal polarity Voc8 and Nat stimuli (Figure 2B), using the new template for ρENV_AN_ to calculate the mean change from ρENV_AN_ to ρENV_MOC_ actually increased the benefits of efferent feedback to envelope encoding for Voc8 stimuli (as well as increased disbenefits to masked stimuli) (S3 Figure B). As discussed in Lines 638-643, this is due to the fact that we are specifically measuring the neural component associated with the stimulus envelope (Joris *et al.*, 2006; Heinz and Swmainthan, 2009). As a result, it is necessary to compare both the neural responses to the normal and polarity-inverted stimuli before concluding whether neural envelopes are more similar for Nat_ANonly_ Vs Voc8_ANonly_ or Nat_MOC_ Vs. Voc_ANonly_.

**Reviewer 4-major comment 6.** Figure 1 D and text: Also here I am confused as to whether ρENVAN on the x-axis uses (a) the corresponding natural or (b) always MOC natural as reference. In case of (a) you can safely conclude that adding MOC has an effect on envelope similarity. In case of (b) you cannot because clearly the MOC simulations will be more similar to the MOC natural than the no-MOC simulations, especially for the VOC conditions. So in case you use (b), the sentence "with largest enhancements observed for words with the lowest ρENV values in the absence of MOC reflect" appears trivial and a consequence of always using MOC natural as the reference for both MOC and no-MOC manipulated speech conditions.

*Response to Reviewer 4-major comment 6:* Although we agree that where values of ρENV_AN_ are already close to 1 for Voc8 stimuli, it is difficult for the MOC reflex to improve envelope encoding further (hence explaining the smaller improvements for larger initial values of ρENV_AN_); the larger enhancements observed for Voc8 stimuli with the lowest ρENV_AN_ scores is evident independent of the control template used to calculate ρENV_AN_ (S3 Figure A). In fact, an inverse relationship between ρENV_AN_ and mean change in ρENV_AN_ to ρENV_MOC_ is observed for both noise-vocoded (i.e., larger benefits for lower ρENV_AN_ values) and masked stimuli (i.e., larger disbenefits for lower ρENV_AN_ values) when AN responses to the Nat_ANonly_ condition are used as the control template to calculate ρENV_AN_ (S3 Figure A). As a result, we have kept the sentence quoted in the results but highlight these new control results (Lines 328-335).

**Reviewer 4-major comment 7.** Model Simulations: To better integrate the ABR results into the resulting final proposed mechanistic MOC reflex model, it would be worthwhile to also simulate a proxi for ABRs with the Meddis model. You can sum up the simulated AN responses across fibers and CFs in the to evaluate how the click ABR would be affected with and without MOC, and see how these alterations correspond to the amplitude changes you observed experimentally. Of course you'd only have a reliable wave-I simulation from that model, but you could add a basic-non gain CN/IC model to simulate the ABR wave-III and V as well (e.g. Nelson and Carney, 2005, Verhulst et al., 2018). It is not problematic to me if you decide not to pursue ABR simulations all the way to the wave-V, but even simulating wave-I would already be informative to better understand the bottom-up MOC strength changes and their effects on expected click ABR amplitude changes (that you also measured).

*Response to Reviewer 4-major comment 7:* We appreciate this idea and believe this may be of interest for further studies however the main aim of our simulations here was to provide a potential explanation for the observed divergence in CEOAE suppression for noise-vocoded and masked stimuli. As a result, we have actually removed the brainstem circuitry from the MAP-BS, using the ‘AN only’ portion alongside a fixed MOC attenuation to simplify and strengthen the emphasis of our simulations (thus bringing them into line with Reviewer 3’s general comment about the model). While we could simulate a wave 1 response from our expanded AN simulations, we do not have equivalent electrophysiological measures for comparison (we measured the amplitudes of waves III (likely cochlear nucleus) and V (likely lateral leminiscus/inferior colliculus). Therefore, any conclusion we make would be indirect and provide no more insight than a number of studies that have demonstrated reduced AN output when the MOC reflex is directly activated in animal models.

**Reviewer 4-major comment 8:** Model Simulations/Data interpretations: On the one hand you state in L671 that you analysed the rms suppression of CEOAE only in the 1-2 kHz region because that is where the MOC reflexes are strongest. On the other hand, you consider envelope tracking as a measure of speech coding and study effects of on-CF MOC simulations. Does this not bias your interpretation, i.e. you simulate MOC effects for speech/noise frequencies outside the 1-2 kHz region as well, where no MOC innervation is expected based on CEOAEs. I am assuming that speech envelope tracking parameters mostly gets their contributions from high-frequency coding (i.e. from ANFs that do envelope tracking with CFs above the phase-locking limit), how do you reconcile these two aspects, and minimize bias on your results interpretation?

*Response to Reviewer 4-major comment 8:* Thank you for this comment. We selected the 1-2kHz band to measure CEOAE suppression by the MOC reflex as it has been shown in numerous studies to provide the largest and most consistent changes across subjects (Hood et al., 1996; Mertes and Goodman, 2015). This does not however indicate that suppression of the cochlear gain is restricted to these frequencies for a couple of reasons: 1. The frequency specificity of CEOAEs is not precise, relying on spatially-distributed irregularities along the basilar membrane (that vary greatly between individuals) for the linear reflections that constitute CEOAEs. Therefore the energy in the 1-2kHz CEOAE band does not necessarily originate solely from the equivalent tonotopic region in the cochlea. This will be especially true at high level intensities (such as the 75dBA used here) where significant energy from nonlinear distortions distant to the 1-2kHz region will likely contribute (Yates and Withnell, 1998); 2. MOC fibers innervate the cochleae in a broad and non-frequency-specific manner (Brown, 2014; Liberman and Liberman, 2019). In conjunction with the broadband click stimuli we used, the MOC reflex will likely be acting along the length of the cochleae when suppression is observed in the 1-2kHz band. We therefore consider suppression of the cochlear gain in the 1-2kHz band as a suitable and consistent marker for MOC reflex activity across the entire cochlea. Given the importance of conveying this argument to our readers, we have included a summary of these points in the Methods (Lines 841-849).

As regards the auditory periphery model, our original simulations were restricted to the 1-2kHz frequency range and therefore only explored the MOC reflex’s effects on envelope encoding within this range (as efferent feedback acted within-channel). Given that our CEOAE results are representative of the MOC reflex’s broader activity along the basilar membrane, we have expanded the range of frequencies (0.1 to 4.5kHz as in Brown et al., 2010) and implemented a fixed (typically 15dB) MOC attenuation of the cochlear gain in all channels (again similar to Brown et al., 2010). These changes allow use to make more general conclusions about how efferent feedback affects envelope encoding without making assumptions about a frequency-specific recruitment of the MOC reflex for different words. Despite these changes, the results of our new simulations demonstrate similar stimulus-dependent effects of adding efferent feedback in both high (>1.5kHz) and low (<1.5kHz) frequencies bands (Figure 2 E and F). While envelope encoding is particularly important in high frequency channels, its importance should not be wholly negated at lower frequencies where recent studies have shown its influence both on binaural localization and speech tracking (Ardoint et al., 2011; Dietz et al, 2013).

**Reviewer 4-minor spelling 12**: Figure 2E "CEOAE inhibition" => use a similar nomenclature in Fig 1 and 2 when referring to the CEOAEs.

*Response to Reviewer 4-minor spelling 12:* Corrected in both figures.

**Reviewer 4-major comment 9**: When recording simultaneous EEG and OAEs, it is possible to generate stimulus artifacts in the EEG traces caused by the ER-10B OAE mic. Did you observe such artifacts, and how did you ensure they had no confounding effect on your ABR amplitudes?

*Response to Reviewer 4-major comment 9:* Thanks for pointing this out. Click artifacts can be observed in the unfiltered EEG recordings (between 0 (click onset)-1msec), however, the data was band-pass filtered between 200-3000 Hz (resulting in small “bump” at click onset for ABR waveform in Figure 1B). Because stimulus artifacts were always observed between 0-1msec and our components of interest between 3-6 msecs, no confounding effects were expected.

**Reviewer 4-major comment 10**: Did you only use only one observer to peak-pick your ABR traces (unusual in audiological studies), and did these amplitudes correspond to wave-III and V peaks to noise-floor or positive peak to negative peak amplitudes?

*Response to Reviewer 4-major comment 10:* ABR waveforms obtained with the weighted-averaging method were visually analysed by the first author (peak-pick) with the assistance of 2 lab members (not listed as authors) (Line 888). As shown in Figure 1B (ABR example recording), the weighted-averaging method calculates the noise floor and we only selected components with a positive SNR when compared to the noise floor.

**Reviewer 4-major comment 11**: How did you minimize the effects of individual CEOAE/MOC strength on your recordings? In our own recordings, we tend to observe that people with strong CEOAEs shows the most suppression. This is not unusual because they have the most gain in their CEOAEs to begin with. Could individual differences in CEOAE amplitude and MOC strength have had an impact on your results?

**Response to Reviewer 4-major comment 11**: We have included new S10 Figure with examples of individual CEOAE suppression data. As observed, we indeed had participants with large CEOAE suppression (subject 4 and subject 20, Active Voc12, S10 Figure), however they were statistically defined as outliers (statistical test for normal distributions) and therefore not included in our statistical analysis. As suggested by Mishra & Lutman 2014; Mertes & Goodman 2016, selecting CEOAEs with high SNR reduces both intra and inter individual variability. As shown in S10 Figure, subjects labelled SNR, had lower than +6 dB SNR in their CEOAE recordings therefore were not included in our statistical analysis. We foresee that our thresholding of data based on SNR and subsequent removal of outliers has made our CEOAE results much less susceptible to obvious differences in individual MOC reflex strength.

***References for reviewer 4 comments***

Giraud, Anne Lise, et al. "Auditory efferents involved in speech-in-noise intelligibility." Neuroreport 8.7 (1997): 1779-1783.

Mertes, Ian B., Kristin M. Johnson, and Zoë A. Dinger. "Olivocochlear efferent contributions to speech-in-noise recognition across signal-to-noise ratios." The Journal of the Acoustical Society of America 145.3 (2019): 1529-1540.

Joris PX, Louage DH, Cardoen L, van der Heijden M. Correlation index: a new metric to quantify temporal coding. Hear Res. 2006;216–217: 19–30.

Heinz MG, Swaminathan J. Quantifying envelope and fine-structure coding in auditory nerve responses to chimaeric speech. J Assoc Res Otolaryngol. 2009;10: 407–423.

Hood LJ, Berlin CI, Hurley A, Cecola RP, Bell B. Contralateral suppression of transient-evoked otoacoustic emissions in humans: intensity effects. Hear Res. 1996;101: 113–118.

Mertes IB, Goodman SS. Within- and Across-Subject Variability of Repeated Measurements of Medial Olivocochlear-Induced Changes in Transient-Evoked Otoacoustic Emissions. Ear Hear. 2016;37: e72-84.

Yates GK, Withnell RH. The role of intermodulation distortion in transient-evoked otoacoustic emissions. Hear Res. 1999;136: 49–64.

Brown GJ, Ferry RT, Meddis R. A computer model of auditory efferent suppression: implications for the recognition of speech in noise. J Acoust Soc Am. 2010;127: 943–954.

Liberman LD, Liberman MC. Cochlear Efferent Innervation Is Sparse in Humans and Decreases with Age. J Neurosci. 2019;39: 9560–9569.

Ardoint, Marine, et al. "Importance of temporal-envelope speech cues in different spectral regions." The Journal of the Acoustical Society of America 130.2 (2011): EL115-EL121.Dietz et al, 2013

Dietz, Mathias, et al. "Emphasis of spatial cues in the temporal fine structure during the rising segments of amplitude-modulated sounds." Proceedings of the National Academy of Sciences 110.37 (2013): 15151-15156.

Mishra SK, Lutman ME. Top-down influences of the medial olivocochlear efferent system in speech perception in noise. PLoS One. 2014;9: e85756.
