# Supplementary material for: Understanding degraded speech leads to perceptual gating of a brainstem reflex in human listeners
Source: PLoS Biol. 2021 Oct 20;19(10):e3001439. doi: 10.1371/journal.pbio.3001439 (PMC8559948; doi:10.1371/journal.pbio.3001439)
Supplement: Attachment — Submitted filename: Response-to-Reviewers.docx [file pbio.3001439.s012.docx]

Reviewer remarks:

Reviewer #1: The authors have considered each and every one of my comments and have addressed all of them adequately. The revised manuscript is much clearer and stronger. The study is very interesting and timely. It will be of interest to hearing scientists and audiologists. It should be published pending the following comments.

General comment
One general comment is that the notion that the MOC reflex can help understanding vocoded speech because it enhances the amplitude modulations in speech was suggested by Lopez-Poveda et al. (2016). Indeed, it is explicitly shown in the insets of Fig. 2 of a more recent study by the same group (Lopez-Poveda et al., 2020, Ear and Hearing). Of course, their conclusions are based on the behavior of a MOC-inspired sound-coding strategy for cochlear implants. It is nice that the present study provides experimental support to that notion also for normal-hearing listeners. What I find puzzling is that Lopez-Poveda et al. (2020) found that the effect and benefit apply to (vocoded) speech in quiet as well as in noise, while the present study find the effect and benefits to apply only in quiet. I would ask the authors to consider highlighting and discussing the issue briefly in their manuscript.

Response: Thanks for this suggestion, we have added a short paragraph in the final section of the Discussion “**Implications for hearing-impaired listeners**” where we briefly discuss Lopez-Poveda et al., 2020 in relation to our own Results. Lines 736-748.

Minor comments
L107. Remove the comma after "different kinds,".

*Authors’ Response:* Edited

L152-153. Do whiskers really represent interquartile range (IQR=q3-q1)? Please double check because this does not seem to be the case for some boxplots, e.g., Natural and Voc16 in the left panel.

*Authors’ Response:* Thanks for noticing this. This is indeed an error. We have edited this section as follows: “whiskers denote the upper (q3+1.5*IQR) and lower bounds (q1-1.5*IQR) of the data where IQR is the interquartile range calculated as (q3-q1).”

L751. Delete the blank space in "to increase ."
*Authors’ Response:* Edited

L775. Delete the comma in "each time, they heard".

*Authors’ Response:* Edited

L793. Delete the blank space in "stop/ non-stop".

*Authors’ Response:* Edited

L812-813. Replace the full stop with a comma in "applied to stimuli. Figure 1C".

*Authors’ Response:* Edited

Figure 2D. Please double check the titles for the panels; they seem incorrect. Specifically it makes no sense to write "Nat_MOC vs Nat_MOC" or "VOC_AN vs VOC_AN".

*Authors Response:* Thank you for this comment. While single trials are technically being correlated against other trials of the same provenance (i.e. Nat_MOC vs Nat_MOC or VOC_AN vs VOC_AN) in Fig 2D’s Shuffled-AutoCorrelograms and Cross-Polarity Correlograms, we agree that this may be confusing to the reader and makes the figure appear crowded. We have therefore edited Figure 2 to read: “Nat_MOC”. “VOC_MOC” and “Nat_AN/VOC_AN” (similar to titles in Fig 1 of Heinz and Swaminathan, 2009).

Figure 3A. Correct the ordinate label in the right-top panel; it reads "nplitude" where it should read "Amplitude".
*Authors’ Response:* Thanks for noticing this. We have edited Figure 3. Now it reads “amplitude” for all variables.

Reviewer #4: Revision:
Understanding degraded speech leads to perceptual gating of a brainstem reflex in human listeners.
Hernandez-Perez, Mikiel-Hunter et al.

I would like to congratulate the authors on their revised manuscript. It follows a unique whole-systems approach to unravel the functional role of the MOC reflex in (degraded) speech perception, a topic that is both timely and important. I appreciate the careful consideration of the reviewer comments/suggestions and subsequent additional analyses, which substantially improved the clarity of the manuscript and led to a stronger scientific argumentation. The editor asked me to also evaluate rebuttal comments to R2, and I can confirm that you addressed both our raised points satisfactory. I have one comment left that you may wish to consider in your final submitted version (no need for another rebuttal on my behalf).

L233: "We conclude from this that the differences observed in cochlear gain and auditory brainstem/midbrain activity can be attributed to the specific form of speech degradation. Together with the effect on CEOAEs, our data suggest that the magnitude of auditory midbrain activity for the different speech manipulations reflects cochlear output. While this is evident for both listening conditions in masked speech, the similarity of ABR amplitudes in the midbrain for active and passive listening of noise-vocoded stimuli is indicative of feed-forward amplification that compensates for reduced cochlear gain during active listening"

I understand how you get to your conclusion, but I find the direct comparison between CEOAE and ABR suppression as motivator for the "feed-forward amplification" explanation a bit tricky. It is a possibility, but you need to also consider that ABRs and CEOAEs reflect activity from somewhat different cochlear regions. Dominant CEOAE energy in 1-2 kHz, whereas regular click ABRs mostly reflect summed activity of the higher frequency regions (3-8 kHz, Abdala & Folsom 1995, Don & Eggermont 1978). Can you be sure that this latter aspect had no influence on your interpretation of results?

*Authors’ Response:* Thanks for this comment. We have added the following rationale the Results section “**Auditory brainstem activity reflects changes in cochlear gain when listening to speech-in- noise**”(Lines 219-222): “Click-evoked ABRs largely reflect summed activity of higher-frequency regions of the cochlea (3-8 kHz, [54,55]). However, as CEOAE suppression in the 1-2kHz band is used here as a marker for MOC reflex activity across the entire length of the cochlea (see Methods), we can relate observed changes in cochlear gain to amplitudes of ABR waves.”

In the Methods section (Lines 859-869) we explain the following: “The averaged RMS magnitudes of CEOAE signals (Figure 1B) were analysed between 1-2 kHz given maximal MOC effects in this frequency band [46,172]. The energy in the 1-2kHz CEOAE band does not necessarily originate solely from the equivalent tonotopic region in the cochlea, especially at high level intensities (such as the 75 dB p-p used here), where significant energy from nonlinear distortions distant to the 1-2 kHz region will likely contribute [173–175]. Given the broadband nature of the click stimuli and the sparse but non-frequency specific nature of MOC innervation of the cochleae [147,148], the MOC reflex will be acting along the length of the cochleae when suppression is observed in the 1-2 kHz band. We therefore consider suppression of the cochlear gain in the 1-2 kHz band as a suitable and consistent marker for MOC reflex activity across the entire cochlea”.

L372-374: consider rephrasing for clarity

*Authors’ Response:* We have edited these lines accordingly.

References

Analysis of the click-evoked brainstem potentials in man using high-pass noise masking
M. Don, and J. J. Eggermont The Journal of the Acoustical Society of America 63, 1084 (1978); doi: 10.1121/1.381816

Frequency contribution to the click-evoked auditory brain-stem response in human adults and infants. Carolina Abdala, and Richard C. Folsom. The Journal of the Acoustical Society of America 97, 2394 (1995); doi: 10.1121/1.411961 View online: [https://doi.org/10.1121/1.411961](https://protect-au.mimecast.com/s/yiFuCjZ12Rf7EJlMhjDPsx?domain=doi.org)
